# Development and validation of a HPLC method for therapeutic monitoring of brivaracetam

**DOI:** 10.11613/BM.2026.020702

**Published:** 2026-04-15

**Authors:** Sven Komljenović, Sara Zovko, Andreja Bujan Kovač, Željka Petelin Gadže, Miranda Sertić, Mila Lovrić

**Affiliations:** 1 Department of Laboratory Diagnostics, University Hospital Centre Zagreb, Zagreb, Croatia; 2 Department of Laboratory Diagnostics, University Clinical Hospital Mostar, Mostar, Bosnia and Herzegovina; 3 Department of Neurology, University Hospital Centre Zagreb, Referral Centre of the Ministry of Health of the Republic of Croatia for Epilepsy, Affiliated Member of the ERN EpiCARE, Zagreb, Croatia; 4 School of Medicine, University of Zagreb, Zagreb, Croatia; 5 Faculty of Pharmacy and Biochemistry, University of Zagreb, Zagreb, Croatia

**Keywords:** method validation, liquid chromatography, brivaracetam, therapeutic drug monitoring

## Abstract

**Introduction:**

Brivaracetam belongs to antiepileptic drugs and due to the interindividual variability, therapeutic drug monitoring is recommended, especially when used in polytherapy and in patients with altered pharmacokinetics. The aim of this study is to develop and validate a high-performance liquid chromatography (HPLC) method with a diode array detector (HPLC-DAD) that is suitable for therapeutic monitoring of brivaracetam and assess method’s greenness.

**Materials and methods:**

High-performance liquid chromatography method was validated according to the International Council for Harmonisation of Technical Requirements for Pharmaceuticals for Human Use (ICH) and Clinical and Laboratory Standards Institute (CLSI) guidelines. Preparation of serum samples was performed by liquid–liquid extraction using ethyl acetate:hexane (1:1) with chloramphenicol as internal standard. Separation was achieved using a 5 µm 4.6 x 250 mm C18 Shim-pack GIST column with the mobile phase consisting of a 50 mM phosphate buffer (pH = 4.5) and organic mixture of acetonitrile and methanol (8/3; v/v). Brivaracetam is quantified at 210 nm. AGREEprep was utilized for the method’s greenness scoring.

**Results:**

The developed HPLC-DAD method is linear over a concentration range of 0.25-20.00 µmol/L. Repeatability was lower than 7.20%, intermediate precision was lower than 8.30%, and within-laboratory precision was lower than 10.50%, except for the values at the lower limit of quantification (0.25 µmol/L) which were still below 20.00%. Bias was lower than ± 10.60%. The method displays very high specificity at 210 nm. The result for method greenness was 0.4.

**Conclusions:**

The validation parameters were within the acceptance criteria of the ICH and CLSI guidelines. The developed method is simple, selective, reproducible, more sensitive than other published HPLC methods and suitable for the therapeutic drug monitoring of brivaracetam.

## Graphical abstract

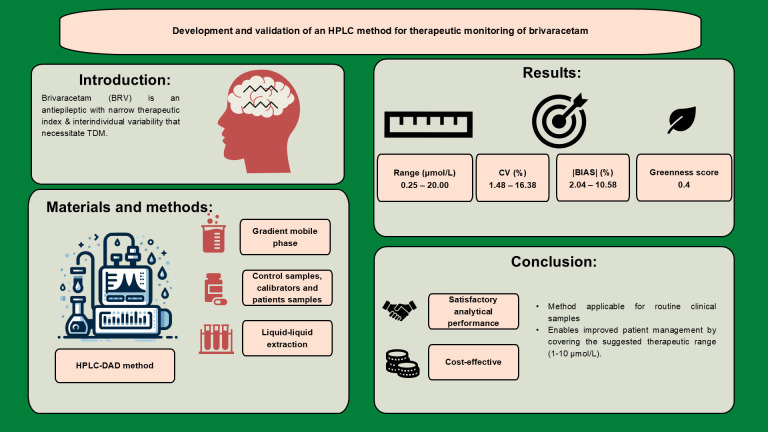

## Highlights

• A novel chromatography method developed for brivaracetam monitoring

• Satisfactory analytical characteristics, cost-effective and accessible

• Method greenness presented for environmental impact and future method development

• Improved laboratory approach and contribution for epilepsy patients

## Introduction

Epilepsy is a chronic neurological disorder that affects about 50 million people worldwide, and even though there are numerous anticonvulsant drugs with different mechanisms of action for the treatment of epilepsy, one of the main challenges is drug resistance in patients, which affects about 30% of them (*1*, *2*). Brivaracetam (BRV; UCB 34714; 2-(2-oxo-4-propylpyrrolidin-1-yl) butanamide) is a third generation of antiepileptic drug. In the European Union, the European Medicines Agency has approved BRV as an adjunctive therapy for focal (partial onset) seizures, with or without secondary generalization in adults, adolescents and children from 2 years of age. In the United States, the Food and Drug Administration has approved BRV as either monotherapy or adjunctive therapy for partial seizures in patients from one month and older (*3*, *4*). Brivaracetam is available in three different pharmaceutical formulations: tablets, oral solution and intravenous injection solution (*3*).

Like its structural analogue levetiracetam (LEV), BRV acts by binding to synaptic vesicle glycoprotein 2A (SV2A), which is located in the presynaptic membrane and is expressed primarily in the brain, and has an important function in epileptogenesis (*5*, *6*). It is believed that due to its higher lipophilicity (LogD 1.04) compared to LEV (LogD 0.64), BRV passes more easily through the blood-brain barrier and achieves its effect more quickly (*7*). Furthermore, BRV has as much as 15-30 times higher affinity for SV2A than LEV (*8*).

Brivaracetam has linear pharmacokinetics, it is rapidly absorbed from the gastrointestinal tract and is weakly bound to plasma proteins (*9*, *10*). In the liver, it is metabolized mostly by hydrolysis that is not dependent on cytochrome P450 (CYP), but also *via* cytochrome P450, where the enzyme CYP2C19 plays the most important role, and its metabolites are mainly excreted in the urine (*11*, *12*). The most common side effects that occur with the use of BRV are headache, fatigue, somnolence and dizziness (*13*). Brivaracetam can be considered as an alternative replacement for LEV, as overnight switching from LEV to BRV has been shown in clinical practice to be a quick and safe option and is mainly performed due to psychiatric or behavioral side effects associated with LEV (*6*, *14*-*19*).

Due to the narrow therapeutic index and large interindividual variability in BRV elimination rate, therapeutic drug monitoring (TDM) is recommended, especially when used in polytherapy (*20*, *21*). For the determination of BRV by chromatographic methods, several publications are available in the literature, including reverse phase high performance liquid chromatography (RP-HPLC) for the determination of BRV and its stereoisomers and the determination of BRV in pharmaceutical forms (*22*, *23*). Ultra performance liquid chromatography (UPLC) methods have been developed for the determination of impurities and degradation products of BRV, but also for the determination of BRV and other antiepileptic drugs in biological fluids (*24*-*27*). Liquid chromatography tandem mass spectrometry (LC-MS/MS) and a simple high performance liquid chromatography with ultraviolet spectroscopy (HPLC-UV) methods were also used for determination in pharmaceutical forms and biological fluids (*21*, *28*, *29*). LC-MS/MS are sensitive and reliable methods; however, they are too expensive and often unavailable to some researchers. HPLC is considered a cheaper and more readily available method for TDM. Due to the growing clinical need for TDM of BRV, the aim of this study is to develop and verify an HPLC method with a diode array detector (HPLC-DAD) that is cheap, available and suitable for simple quantification of BRV concentration in serum.

Green analytical chemistry (GAC) aims to reduce or eliminate the use of chemicals that are potentially hazardous to the environment or human health. To make chemical processes greener, it is necessary to use less harmful and cleaner chemicals (*30*). Our goal is to develop an environmentally friendly method that will respect the principles of GAC.

## Materials and methods

Method validation for brivaracetam quantification was done by combining International Council for Harmonisation of Technical Requirements for Pharmaceuticals for Human Use (ICH) guidelines from 2022 for method validation in collaboration with European Medical Agency (EMA) and Clinical and Laboratory Standards Institute (CLSI) guidelines (*31*, *32*).

### Chemicals and reagents

The gradient mobile phase was prepared using an organic mixture of HPLC-grade acetonitrile and methanol obtained from Merck (Merck Millipore, Darmstadt, Germany) and a buffer solution prepared by dissolving sodium dihydrogen phosphate monohydrate obtained from Merck (Merck Millipore, Darmstadt, Germany) in HPLC-grade water obtained using NW Ultra-pure Water System (Nirosta, Osijek, Croatia). Chemicals used for extraction were ethyl acetate, hexane, sodium hydroxide (NaOH) obtained from Merck (Merck Millipore, Darmstadt, Germany) and a chloramphenicol standard obtained from Sigma (Sigma-Aldrich, Burlington, USA), used as an internal standard (IS).

## Instruments and chromatography conditions

The analysis was performed using a Prominence HPLC system (Shimadzu Corporation, Kyoto, Japan) with a diode array detector (DAD). Chromatographic separation was achieved using a 5 µm 4.6 x 250 mm C18 Shim-pack GIST column (Shimadzu Corporation, Kyoto, Japan) as the stationary phase, while the mobile phase consists of 2 solutions: the first solution contains a 50 mM sodium dihydrogen phosphate solution (pH = 4.5), and the second an organic mixture of acetonitrile and methanol (ratio 8:3). Efficient chromatographic separation was achieved with a 20-minute analysis by applying a linear gradient mobile phase (from 30 to 60% organic mixture in 16 minutes) until the end of the analysis.

### Calibrators and quality control samples

The calibration curve was constructed using commercially available four-point calibrators from Recipe (RECIPE Chemicals + Instruments GmbH, Munich, Germany). Four points consisted of blank calibrator (matrix without BRV), a 1.33 µmol/L concentration point, a 6.70 µmol/L concentration point and a 19.4 µmol/L concentration point. Commercially available control samples in two levels (RECIPE Chemicals + Instruments GmbH, Munich, Germany) were used to assess precision and accuracy. The lower-concentration control sample was manually diluted with blank serum pool and used to estimate the limit of detection (LOD) and the lower limit of quantification (LLOQ).

### Sample preparation

Samples were prepared using a liquid-liquid extraction (LLE) procedure. A 500 μL aliquot of the sample (patient serum, control, or calibrator) was pipetted into a tube. Subsequently, 25 μL of internal standard (IS) (chloramphenicol, 0.1 mg/mL) and 50 μL of NaOH were added, followed by vortexing. For LLE, 3 mL of an ethyl acetate:hexane (1:1) mixture was added, the tube was capped, vortexed, mixed for 15 minutes on a horizontal shaker and centrifuged to separate the organic and aqueous phases. A 2.5 mL aliquot of the organic phase (upper layer) was transferred to a new tube and evaporated to dryness under air flow. The residue was reconstituted in 150 μL of a mobile phase and transferred to an autosampler vial for chromatographic analysis.

### Selectivity/specificity

The selectivity of the method was evaluated by analyzing 20 random patient samples from residues of serum sample (morning, fasting) of patients treated with another psychotropic drug, but not with BRV. UV spectra were also used to confirm the selectivity of the method.

### Analytical sensitivity

Analytical sensitivity parameters of the method: limit of detection, the lowest concentration that can be detected with statistical significance and lower limit of quantitation, the lowest analyte concentration that can be quantified with a predefined accuracy and precision, were based on a standard deviation of the noise response from blank samples at retention time of BRV. The values obtained from the selectivity/specificity assessment were used. To define LOD and LLOQ, the arithmetic mean of the 20 mentioned samples was used and the triple or tenfold value of the standard deviation was added, respectively. The calculated LLOQ obtained was assessed by other approach based on precision and accuracy, provided that the specified value meets the criterion of coefficient of variation (CV) ≤ 20% (ICH guideline M10 on bioanalytical method validation). The LLOQ confirmation sample was prepared by diluting the control sample with blank serum pool.

### Linearity

The linearity of the method for BRV determination was evaluated using a one-point available commercial calibrator with concentration of 20.00 µmol/L (RECIPE Chemicals + Instruments GmbH, Munich, Germany). Linearity was verified by serial dilution of the calibrator with a blank serum pool to achieve six concentration levels (0.63, 1.25, 2.50, 5.00, 10.00, and 20.00 µmol/L), with a total of 29 measurements performed across the range. The acceptance criterion from the target value was CV ≤ 15%, BIAS ≤ ± 15% while the criterion at the LLOQ was CV ≤ 20%, BIAS ≤ ± 20%.

### Precision and accuracy

Precision and accuracy data were obtained by running control samples in quintuplicate over 5 days. To assess precision, repeatability (Sr), intermediate precision (Sb), and within-laboratory precision (SWL) were calculated along with their corresponding coefficients of variation. The acceptance criterion for the listed parameters was CV ≤ 15%, while the criterion at the limit of quantification was CV ≤ 20% (ICH guideline M10 on bioanalytical method validation). Control samples had declared values of 4.12 and 9.61 µmol/L while at the LLOQ concentrations were diluted with blank serum pool to target values of 0.40 and 0.25 µmol/L.

### Method greenness

To evaluate the environmental and occupational health impact of the method, the AGREEprep software was employed (*33*). This tool quantifies various factors such as sample preparation procedures, chemicals, energy consumption, and safety measures. The resultant score, ranging from 0 (least green) to 1 (greenest), provides a quantitative assessment of the method’s environmental sustainability.

### Clinical application

We evaluated the applicability of the newly developed HPLC-DAD method by processing routine serum samples for TDM of patients with epilepsy on chronic brivaracetam therapy in combination with other antiepileptic drugs. Therapeutic drug monitoring was a part of the patients’ routine clinical management. The clinical study has been positively assessed and approved by the Ethics Committee University Hospital Center Zagreb on March 6, 2023, in Zagreb (Class: 8.1-23/62-4; Number: 02/21 AG). To evaluate therapeutic intervals for BRV, patients’ results from two separate periods were compared to the suggested range of 1.00-10.00 µmol/L. The first period included 50 samples collected from March to October 2023, and the second period also included 50 patients’ samples, collected from May to October 2024. Fasting blood samples, used for therapeutic interval evaluation, were taken in the morning at a 12-hour distance from the evening brivaracetam dose. Blood samples were collected at the Department of Laboratory Diagnostics, University Hospital Center Zagreb, in various Greiner Bio-One VACUETTE serum (Greiner Bio-One, Kremsmünster, Austria) tubes without anticoagulant. The blood samples were rested for a minimum of 30 minutes (depending on the transport time between the sampling site and the central lab) and then centrifuged at 3000 rpm for 5 minutes to separate the serum. The separated serum was immediately frozen at - 20 °C and stored for later analysis, unless the BRV analysis was scheduled for the same day. Samples were kept stored for up to 28 days after collection, after which they were thawed at room temperature for 1 hour and subsequently vortexed before analysis. These storage conditions are consistent with the manufacturer’s specifications for the QC materials and calibrators.

### Statistical analysis

Statistical analysis was performed using Microsoft Excel (Microsoft, Redmond, U.S.) and MedCalc Statistical Software version 23.2.6 (MedCalc Software Ltd., Ostend, Belgium). Normality of distribution was assessed using the Shapiro-Wilk test. Data following a normal distribution are presented as mean ± standard deviation, while non-normally distributed data are expressed as median and interquartile range (IQR). Linearity was evaluated using Weighted Least Squares (WLS) linear regression (1/x^2^ weighting factor) to mitigate the effect of heteroscedasticity. The line equation, slope, intercept, and coefficient of determination (R^2^) were calculated. To further assess the association between theoretical and measured values, Spearman’s rank correlation (r_s_) was applied on the full linearity dataset (N = 29). Comparison of brivaracetam concentrations between the two clinical periods was performed using the Mann-Whitney test. In all analyses, P-values < 0.05 were considered statistically significant.

## Results

### Chromatograms and specificity

**Figure 1:**
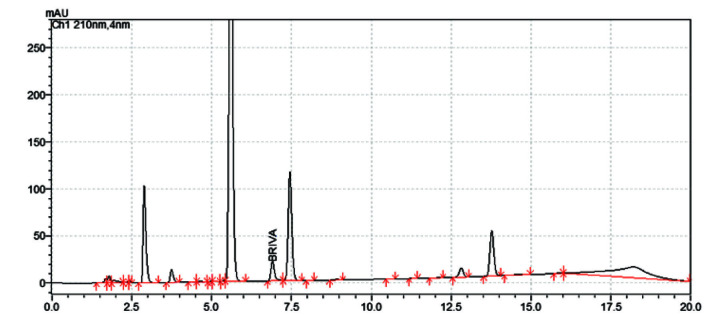
Chromatogram of a patient’s sample with brivaracetam therapy.

**Figure 2:**
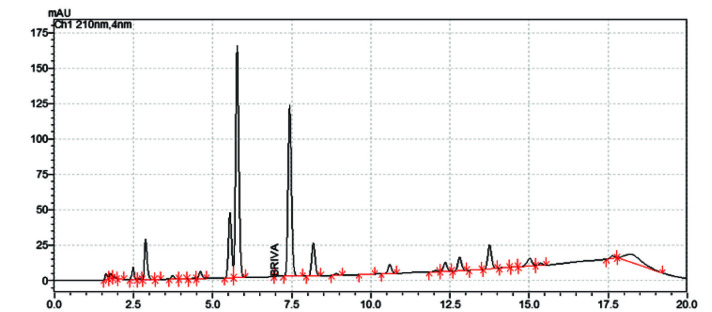
Chromatogram of blank sample demonstrating lack of brivaracetam peak at 6.9 min.

**Figure 3:**
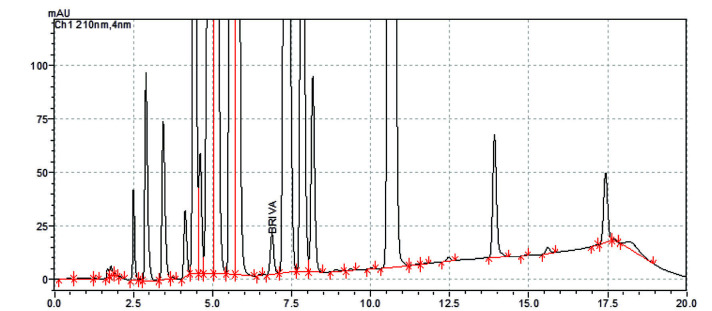
Chromatogram of quality control sample showcasing brivaracetam peak at 6.9 min, observed at 210 nm.

After adjusting the chromatographic conditions, the BRV peak was eluated at the retention time of 6.9 min and detected at 210 nm (Figure 1), free from any overlapping peaks. The method demonstrated high specificity, as injection of the blank serum pool showed no interfering peaks at the retention time of BRV (6.9 min) and at 210 nm (Figure 2). This was further confirmed by the chromatogram analysis of 20 blood samples that did not contain BRV. No interferences on BRV separation were observed after injecting quality control samples containing commonly co-prescribed antiepileptics, their metabolites, and other relevant co-medication drugs (Figure 3).

**Figure 4:**
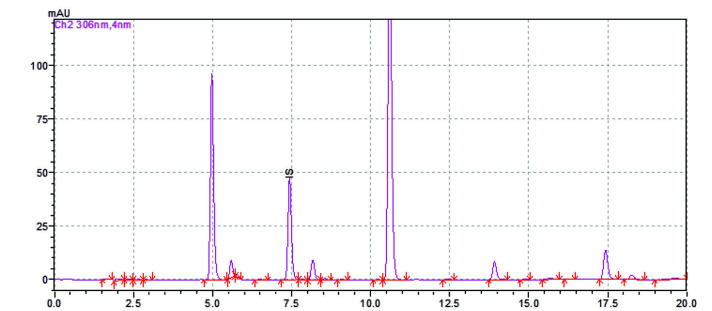
Chromatogram of quality control sample showcasing internal standard peak at 7.5 min, observed at 306 nm.

At the chosen chromatographic conditions, IS has a retention time of 7.5 min and at a wavelength of 210 nm, there is an overlap with the unknown substance from the blank sample pool and QC material. Because of this interference, we chose another wavelength (306 nm) for detection and quantification of IS, where detected substance has no absorption (Figure 4).

### Analytical sensitivity

Analytical sensitivity parameters LOD and LLOQ, calculated from blank sample analyses, were 0.02 µmol/L and 0.05 µmol/L, respectively. However, subsequent validation showed that the LLOQ of 0.05 µmol/L did not meet the criteria for precision and accuracy. Therefore, the working range was extended to 0.25 µmol/L, a level which met the criteria for precision and accuracy.

### Linearity

**Figure 5:**
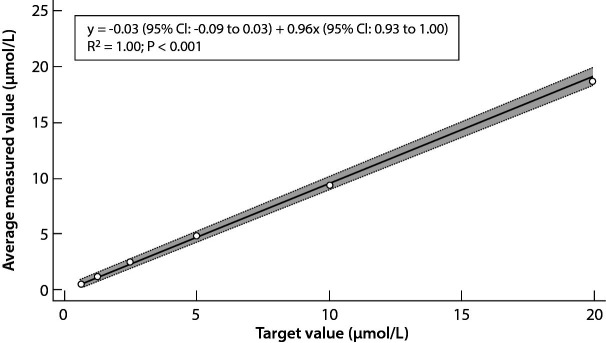
Brivaracetam method linearity. Data points represent the mean of replicates at each level.

The method exhibited satisfactory linearity over the tested range. The regression equation was: y = 0.963 x (95% CI: 0.927 to 0.999, P < 0.001) - 0.032 (95% CI: - 0.088 to 0.025, P = 0.194). The results showed proportional, but no constant difference (Figure 5). The coefficient of determination (R^2^) was 0.9993 (P < 0.001). A strong correlation was confirmed between measured and target values (r_s_ = 0.99, P < 0.001). Coefficients of variation for replicates ranged from 5.89% to 8.98%, and the bias ranged from - 2.10% to - 10.32% at the lowest concentration (Table 1).

**Table 1:** Summary of concentrations, CVs, and BIAS for linearity of an HPLC method for therapeutic monitoring of brivaracetam

**Target value (µmol/L)**	**Average measured value (µmol/L)**	**CV (%)**	**BIAS (%)**
20.00	18.82	5.89	- 5.90
10.00	9.40	6.60	- 5.99
5.00	4.90	5.98	- 2.10
2.5	2.44	6.49	- 2.61
1.25	1.19	8.98	- 5.06
0.63	0.56	8.32	- 10.32
CV - coefficient of variation.			

### Precision and accuracy

Results for repeatability, intermediate precision and intra-laboratory precision were within acceptable limits of 15% (overall) and 20% at the LLOQ (Table 2). As expected, CVs were higher at lower concentrations, consistent with the expected decrease in precision closer to the LLOQ (CV 16.38%). Accuracy results were also satisfactory (BIAS - 10.58 to 4.29%) and showed a similar trend of increasing variability at lower concentrations.

**Table 2:** Repeatability, intermediate precision, intra-laboratory precision and accuracy results of an HPLC method for therapeutic monitoring of brivaracetam

**Target value (µmol/L)**	**Average measured value (µmol/L)**	**Sr (CV%)**	**Sb (CV%)**	**SWL (CV%)**	**BIAS (%)**
4.12	4.20	3.04	6.15	6.73	2.04
9.61	10.02	1.48	5.98	6.13	4.29
0.40	0.38	7.17	8.22	10.42	- 4.01
0.25	0.22	6.57	15.28	16.38	- 10.58
Sr – repeatability. Sb - intermediate precision. SWL - within-laboratory precision.					

### Method greenness

**Figure 6:**
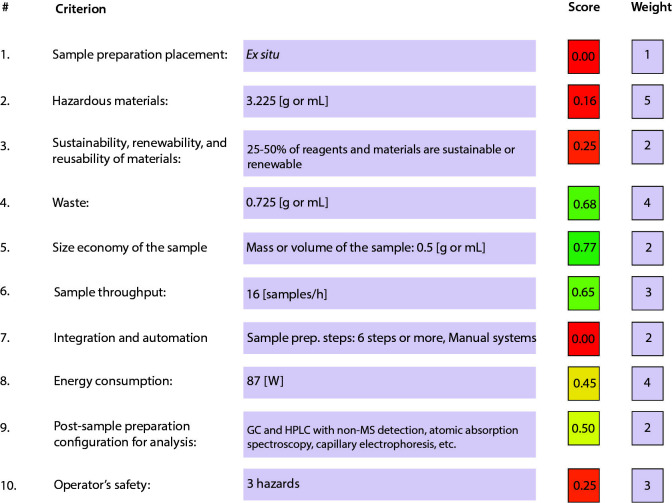
AGREEprep scoreboard showcasing score and weight for each variable in analytical method.

Using the AGREEprep software, a value of 0.4 was obtained (Figure 6). Since strict guidelines for acceptable GAC scores and published papers on HPLC BRV method validation with GAC assessment are rare, we conclude that, under these conditions, the obtained score is acceptable.

### Clinical application

The HPLC method for therapeutic monitoring of brivaracetam was introduced at the request of physicians who prescribed BRV for the treatment of resistant epilepsy. Initial doses were 50 mg twice a day and we used therapeutic range of 2.40-4.30 µmol/L (0.50-0.90 mg/L) which has been suggested in Consensus Guidelines for Therapeutic Drug Monitoring in Neuropsychopharmacology (*34*). After a certain time, the doses were raised to 100 mg twice a day, which required a correction of the therapeutic range. In accordance with clinicians, we implemented another preliminary therapeutic range of 1.00-10.00 µmol/L, which has been proposed based on clinical trials (*35*).

The utility of the suggested therapeutic interval was assessed by comparing results from two periods. In the first period, March-October 2023, 3 out of 50 samples were excluded from calculations due to falsely ordered BRV, resulting in concentrations below the LLOQ. The data from the samples analyzed were not normally distributed (P < 0.001). The median concentration (IQR) was 6.10 (5.70) µmol/L. The percentage of agreement with the therapeutic interval was 85%. In the second period, May–October 2024, all 50 patient samples were used. One sample showed a concentration below the LLOQ, and a value of 0 was used in the calculation. The percentage of agreement in the second period with the therapeutic interval was 70%. The data from the second period showed a normal distribution (P = 0.198). The average concentration was 7.44 ± 3.77 µmol/L. No difference in concentrations between the two periods was observed (P = 0.130).

## Discussion

In this study, an HPLC-DAD method was developed and validated for the determination of BRV concentration in serum. This is particularly important for patients who do not exhibit an adequate response to therapeutic doses, have impaired renal function, or possess an altered CYP2C19 genotype. A diode array detector was employed due to its sufficient sensitivity and specificity in quantifying a variety of drugs. Given the structural similarity between BRV and its analog, LEV, chromatographic conditions were adopted to harmonize the procedure with existing analytical procedures.

The use of chloramphenicol as an internal standard ensured adequate compensation for matrix effects and extraction variations, aligning with ICH recommendations for bioanalytical methods. Furthermore, the choice of LLE proved to be an effective strategy to enhance sensitivity and obtain cleaner extracts without the complexity and costs associated with solid-phase extraction.

The developed method demonstrated satisfactory specificity, where minor interference observed at the retention time of the IS was successfully addressed by wavelength adjustment, without compromising the reliability of quantification.

The initially estimated LLOQ did not fulfill the acceptance criteria for precision and accuracy. Therefore, a higher LLOQ was established to ensure reliable and reproducible measurements, which is essential for TDM. The slight negative bias observed during linearity assessment remained within acceptable limit. Conversely, regression and correlation confirmed linear relationship. The achieved LLOQ and confirmed linearity successfully complement the reported therapeutic range (1.00-10.00 µmol/L). This enables the accurate detection of underdosed and overdosed patients, as well as stratification from non-compliant patients.

Several HPLC and LC-MS/MS methods for the determination of the BRV in biological fluids have been reported in the literature. The developed HPLC-DAD method provides adequate sensitivity for the quantification of BRV concentrations, and the validation parameters obtained in this study are comparable to those reported. The established performance surpassed previously published HPLC method with an LLOQ around 10 µmol/L and is in line with some published LC-MS/MS methods with an LLOQ around 0.50 µmol/L (*25*, *28*, *29*). Although other LC-MS/MS methods demonstrate superior sensitivity (LLOQ around 9 and 3 nmol/L), this enhanced sensitivity was primarily utilized for pharmacokinetic studies or drug abuse monitoring and not for TDM (*24*, *27*). At the similar concentration (2.36 µmol/L), the latter LC-MS/MS method achieved CV ≤ 5.68% and BIAS ≤ - 11.98%, which we found comparable to our results (*27*).

The greenness evaluation indicates that the environmental impact of the method is influenced by the non-automated sample preparation and utilization of hazardous chemicals. Although several methods reported scores around 0.7, they were either developed for BRV measurement in pharmaceutical formulations or utilized different methodologies for TDM (*36*-*38*). The transition to automated systems or commercial kits would likely lower the negative environmental contribution; however, the high investment costs associated with such improvements pose a significant challenge for financially strained facilities.

Clinical application was established through cooperation with physicians who prescribed BRV for the treatment of resistant epilepsy. While BRV concentrations in both monitoring periods were within the suggested therapeutic interval, neither period met the 90% agreement requirement, suggesting that these intervals may require further refinement in clinical practice.

In conclusion, results of the validation of the chromatographic method using a diode array detector displayed that the method is simple to use and exhibits a good signal response. It covers the suggested therapeutic range, significantly improving the clinical approach to patient’s TDM.

## Data Availability

The data generated and analyzed in the presented study are available from the corresponding author on request.
